# Pd NPs supported on halloysite functionalized with Schiff base as an efficient catalyst for Sonogashira reaction

**DOI:** 10.1038/s41598-021-85821-2

**Published:** 2021-03-18

**Authors:** Mansoureh Daraie, Majid M. Heravi, Yalda Rangraz, Zahra Besharati

**Affiliations:** Department of Chemistry, School of Science, Alzahra University, Vanak, Tehran, Iran

**Keywords:** Catalyst synthesis, Heterogeneous catalysis, Green chemistry

## Abstract

A hybrid system was designed and synthesized through reacting modified halloysite (Hal-Cl) with Schiff base (DAB-PC) and applied as catalytic support for anchoring Pd NPs to afford Pd@Hal-DAB-PC catalyst. The resultant material was well identified by various analyses including Fourier transform infrared spectroscopy (FT-IR), X-ray diffraction patterns (XRD), thermogravimetric analysis (TGA), field-emission scanning electron microscopy (FE-SEM), energy-dispersive X-ray spectroscopy (EDS), transmission electron microscopy (TEM), and inductively coupled plasma-optical emission spectrometry (ICP-OES) and revealed outstanding catalytic activity in the Sonogashira reaction in aqueous media. Also, This nanocatalyst was simply collected and recycled up to six runs with a slight drop in efficiency, indicating the durability of Pd@Hal-DAB-PC.

## Introduction

Nowadays, catalysts play a pivotal role in approximately 85–90% of the modern processes in the chemical industries because they can decrease the risk associated with the preparation and utilization of different chemical compounds. Despite this, homogeneous catalysts suffer from shortcomings including considerable difficulties in the separation and recycling and low chemical and thermal stability. Hence, the design and fabrication of metal nanoparticles (MNPs) based-heterogeneous catalysts are highly desirable^1,2^.

The immobilization of the MNPs onto robust supports with large surface area preserves them from agglomeration during the manufacture of the catalyst or in a catalytic reaction and improves their efficiency and stability^3–7^.

Recently, the organic and inorganic nanotubular materials have been widely utilized as catalytic supports due to their outstanding surface area and unique tubular morphology with an empty cavity^8–12^.

In this regard, halloysite (Hal), an octahedral layered aluminosilicate with the formula of Al_2_(OH)_4_Si_2_O_5_·nH_2_O has gained increasing attraction in diverse scientific and industrial fields such as drug delivery, adsorbents, cleaning, and catalysis owing to high chemical and mechanical stability as well as tubular structure. Although physical and chemical features of this naturally occurring clay are similar to kaolin, some characteristics like adjustable surface chemistry and chemical composition on the inner and outer surfaces and various electrical charges make it different from classical ones^13–15^.

Schiff bases have developed as an important research field because of structural diversity and facile preparation from the condensation of carbonyl compounds with primary amines. Moreover, metal complexes of Schiff bases display a diverse range of biological activities and also catalytic activity in different transformations^16–19^.

Among palladium-catalyzed cross-coupling reactions, the Sonogashira cross-coupling which allows the construction of C (sp^2^)-C (sp) bonds by coupling aryl or vinyl halides with terminal alkynes is one of the prominent and practical synthetic methods in organic synthesis^18^. The resultant aryl alkynes and conjugated enynes are extensively utilized in the fabrication of polymers, pharmaceutical compounds, natural products, substituted alkynes, as well as optical materials^21–24^.

**Scheme 1:**
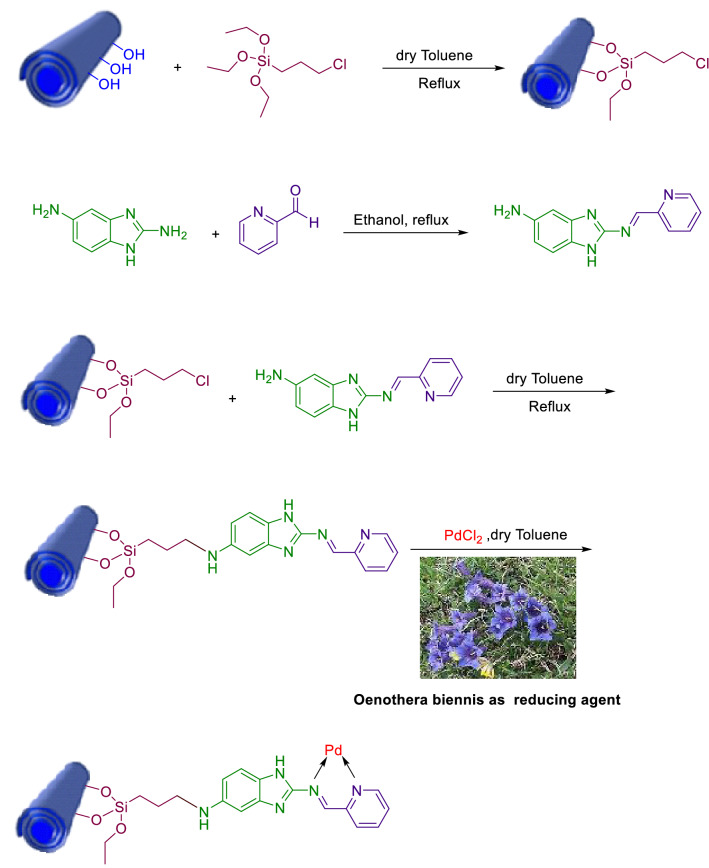
The representation of the fabrication of Pd@ Hal-DAB-PC.

As a part of our efforts towards developing environmentally–friendly catalytic systems for chemical transformations, such as cross-coupling reactions^26–30^, herein, we described the fabrication of a novel heterogeneous catalyst, Pd@Hal-DAB-PC, using a multi-step modification of halloysite and stabilization of Pd NPs (Scheme 1) and its utilization in the Sonogashira reaction.

## Experimental

### Materials and instruments

All the materials utilized for the fabrication of the catalyst and doing Sonogashira reactions, such as Hal, CPTES, 1*H*-benzimidazole-2,5-diamine (DAB), pyridine-2-carbaldehyde (PC), triethylamine, palladium chloride, aryl halides, propargyl alcohol, phenylacetylene, NaOH, K_2_CO_3_, toluene, ethanol, and methanol were obtained from Sigma‐Aldrich and applied as supplied.

The successful synthesis of Pd@Hal-DAB-PC was affirmed using different techniques involving FT-IR, XRD, TGA, FE-SEM, EDX, ICP, and TEM.

In catalytic characterizations, FT-IR spectra were recorded by using PERKIN-ELMER-Spectrum 65 instrument. SEM analysis was carried out over a FESEM-TESCAN MIRA3 microscope with attached EDX (TSCAN). XRD was recorded using Cu Kα radiation (wavelength of 1.78897 Angstrom, 40 keV and 40 Ma). Transmission electron microscope (TEM) images were recorded with CM30300 Kv field emission transmission electron microscope. To perform ICP analysis, an ICP analyzer (Vista-pro, Varian) was employed. A Mettler Toledo TGA instrument was employed for accomplishing TGA, using nitrogen atmosphere. NMR spectra of the organic compounds were measured with a BRUKER spectrometer.

### Fabrication of Pd@Hal-DAB-PC

#### Fabrication of Hal‐Cl (Hal modification with (3-chloropropyl)triethoxysilane (CPTES))

First, 1.5 g of Hal was ultrasonically spread in 40 ml of dry toluene for 30 min. In the following, 1.5 g of CPTES was dropwise injected into the above suspension and next refluxed under flowing N_2_ for 24 h. Finally, the resulting mixture was filtered and repeatedly rinsed with toluene. After drying in an oven at 80 °C for 24 h, Hal was decorated with CPTES.

#### Synthesis of the Schiff base ligand (DAB-PC)

0.108 g of Pyridine-2-carbaldehyde (1.0 mmol) was reacted with 0.147 g of benzimidazole-2,5-diamine (1.0 mmol) in EtOH for 4 h at reflux temperature. The resulting pale-yellow solid was separated by filtration. After drying, (2-((pyridine-2-ylmethylene)amino)-1H-benzo[d]imidazole 5-amine) was provided as a pale yellow powder^31^.

#### Incorporation of Schiff base: synthesis of Hal-DAB-PC

Initially, 1.5 g of Hal‐Cl was spread in the dry toluene by sonication. In the following, 1.5 g of Schiff base was added to the mentioned suspension in the attendance of 2 ml of triethylamine as a catalyst and subsequently, heated at 110 °C for 1 day. The resultant solid was collected after completing the reaction, rinsed with dry toluene several times, and next dried at 100 °C for 24 h.

#### Preparations of Oenothera biennis extract

Firstly, 2 g of Oenothera biennis which were collected from Kurdistan, Iran, ground in a porcelain mortar. After that, the obtained powder was thoroughly mixed with 100 ml of deionized water and heated for 1 h at 80 °C. The resulting solid was isolated using facile filtration after cooling the mixture, and the extract was achieved.

#### Immobilization of Pd nanoparticles on Hal-DAB-PC by using Oenothera biennis extract as a reducing agent

For the immobilization of Pd NPs, 1.2 g of Hal-DAB-PC was added to 15 ml of dry toluene that containing 0.02 g palladium chloride was, followed by stirring for 12 h at ambient temperature. In the next step, 10 ml of Oenothera biennis extract was added and the resultant mixture was allowed to stir for another 5 h. The final product was isolated, rinsed with MeOH (three times), and then dried in an oven at 60 °C for 24 h to provide Pd@ Hal-DAB-PC (Scheme 1).

#### General method for Sonogashira reaction

**Scheme 2:**
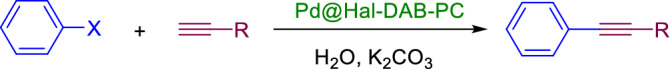
Sonogashira reaction catalyzed by Pd@Hal-DAB-PC.

To a mixture of aryl halide (1 mmol) and terminal alkyne (1.2 mmol) in H_2_O as the solvent, Pd@Hal-DAB-PC (10 mol%) and K_2_CO_3_ (3 mmol) were added and the resulting mixture was heated at 100 °C for the desired time (Scheme 2). The proceeding of the reaction was followed by TLC. After finishing the reaction, the Pd@Hal-DAB-PC was isolated, washing done with EtOH, dried, and then reused for the next run under similar conditions. The organic layer was extracted via diethyl ether and purified using column chromatography [*n*-hexane/ethyl acetate (4:1)] to gain the respective product.

## Result and discussion

### Characterization of catalyst

**Figure 1:**
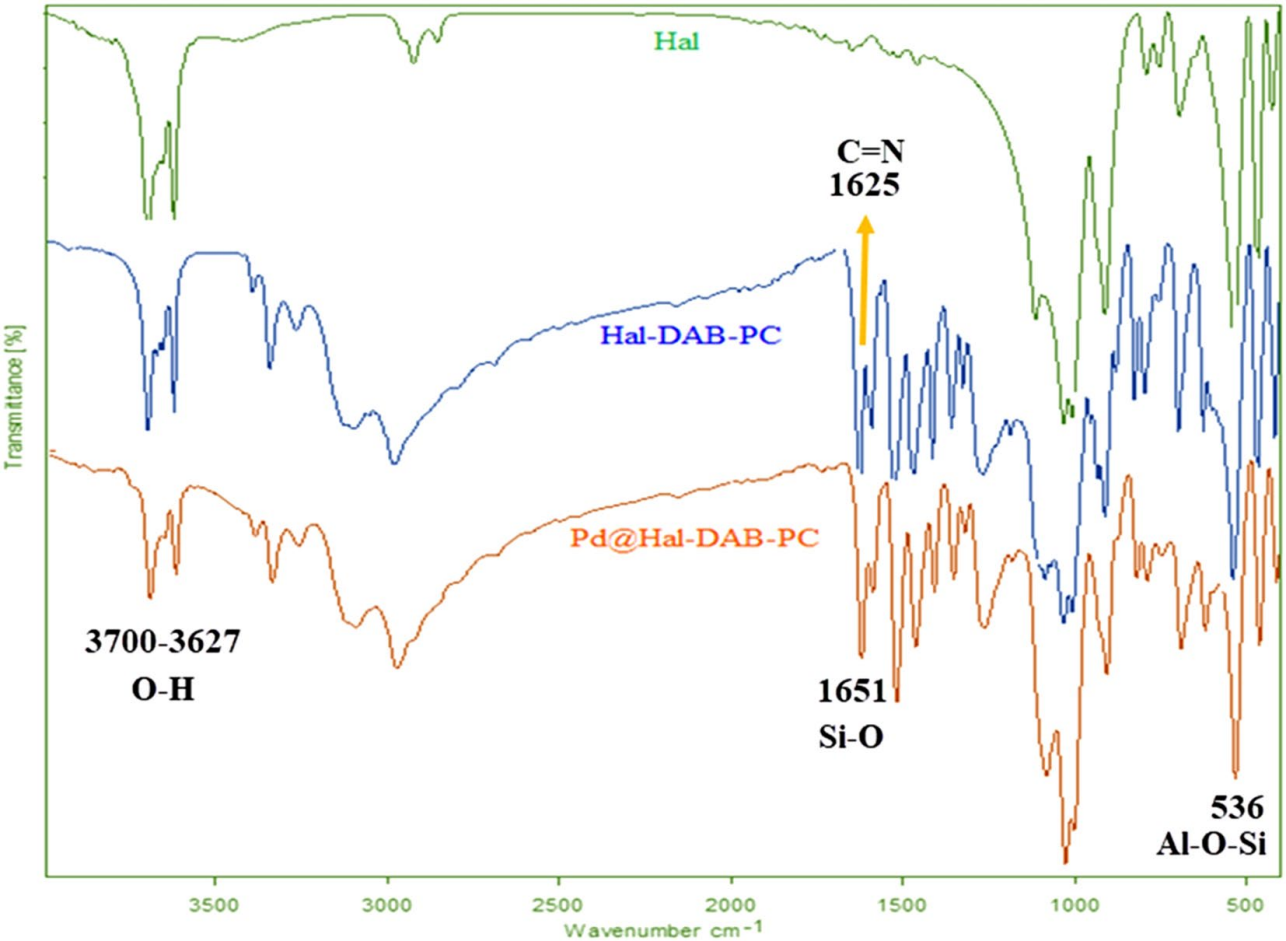
The FTIR spectrums of Hal, Hal-DAB-PC, and Pd@Hal-DAB-PC.

The FT-IR spectra of Hal, Hal-DAB-PC, and Pd@Hal-DAB-PC are illustrated in Fig. 1. In the FT-IR spectrum of Hal, the prominent absorption peaks at 536, 1651, and 3627–3700 are ascribed to the stretching vibrations of Al–O–Si, Si–O, and inner -OH groups, respectively^32^. The emergence of a new absorption peak at 1625 cm^−1^ corresponding to stretching vibration of C=N in the FT-IR spectrum of Hal-DAB-PC affirms the successful synthesis of Schiff base. There is not an obvious change in the spectrum of Pd@Hal-DAB-PC, suggesting that the immobilization of Pd does not influence the distinctive peaks of Hal-DAB-PC.

**Figure 2:**
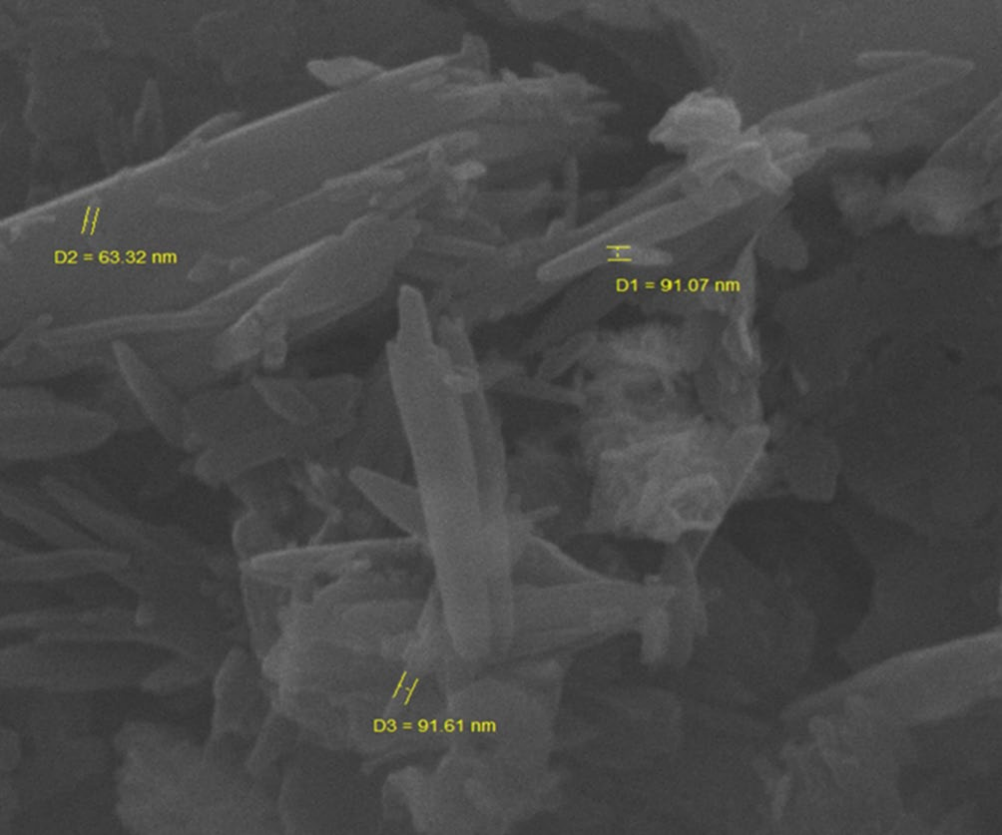
SEM image of Pd@Hal-DAB-PC.

**Figure 3:**
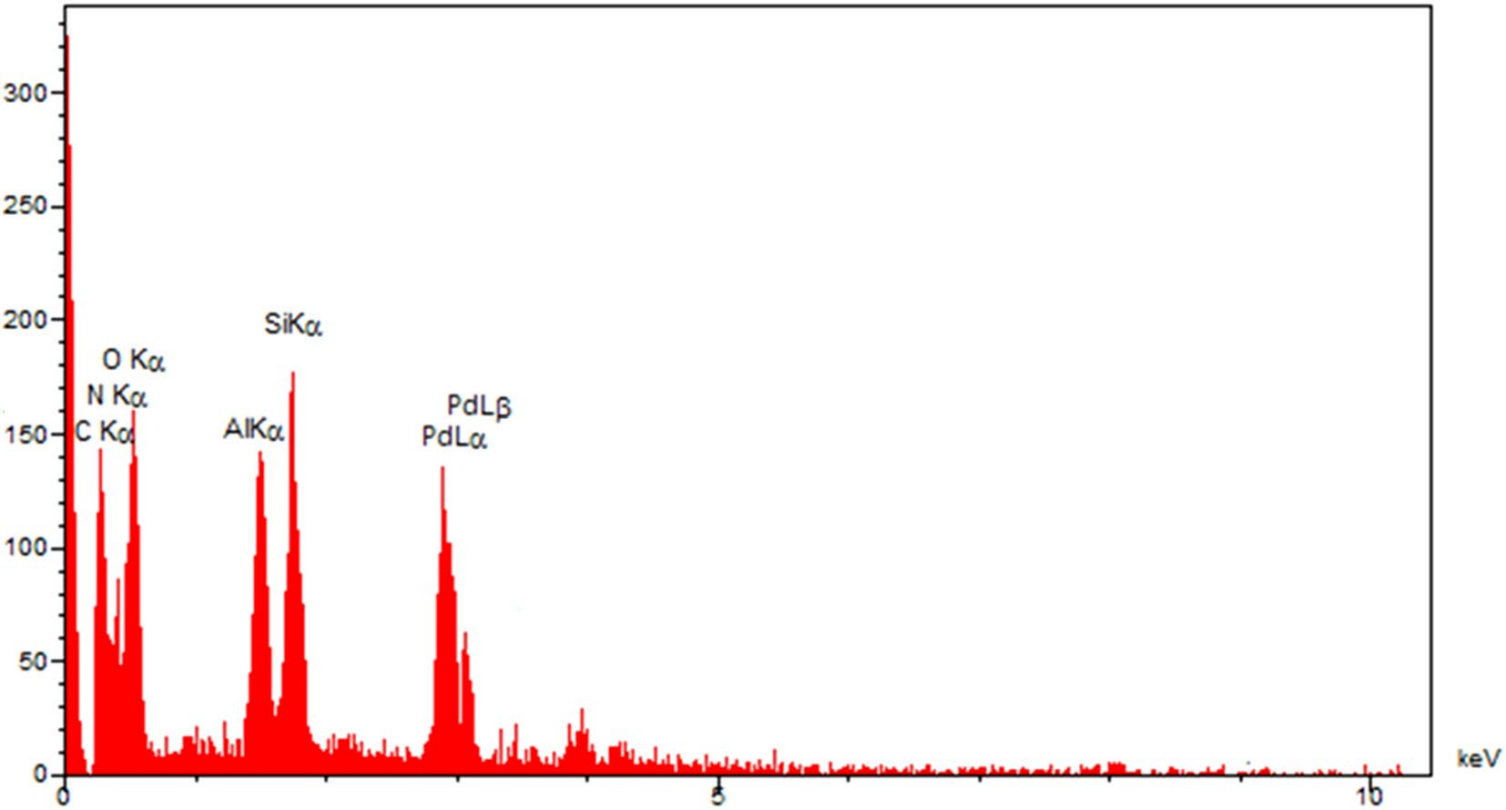
EDX spectrum of Pd@Hal-DAB-PC.

The morphology, as well as the chemical composition of Pd@Hal-DAB-PC, were surveyed by FE-SEM and EDS analyses (Figs. 2, 3). The SEM image of the catalyst exhibits that the tubular morphology of Hal remained unchanged after modification with Schiff base and incorporation of Pd NPs. Also, the EDX spectrum of the Pd@Hal-DAB-PC demonstrates the presence of Al, Si, and O elements which are ascribed to the Hal structure. Apart from these elements, the observation of the peaks of C, N, and Pd authenticates the attendance of Schiff base as well as Pd NPs in the final structure of Pd@Hal-DAB-PC.

Also, the EDX spectrum of the Pd@Hal-DAB-PC demonstrates the presence of Al, Si, and O elements which are ascribed to the Hal structure. Apart from these elements, the observation of the peaks of C, N, and Pd authenticates the attendance of Schiff base as well as Pd NPs in the final structure of Pd@Hal-DAB-PC.

**Figure 4:**
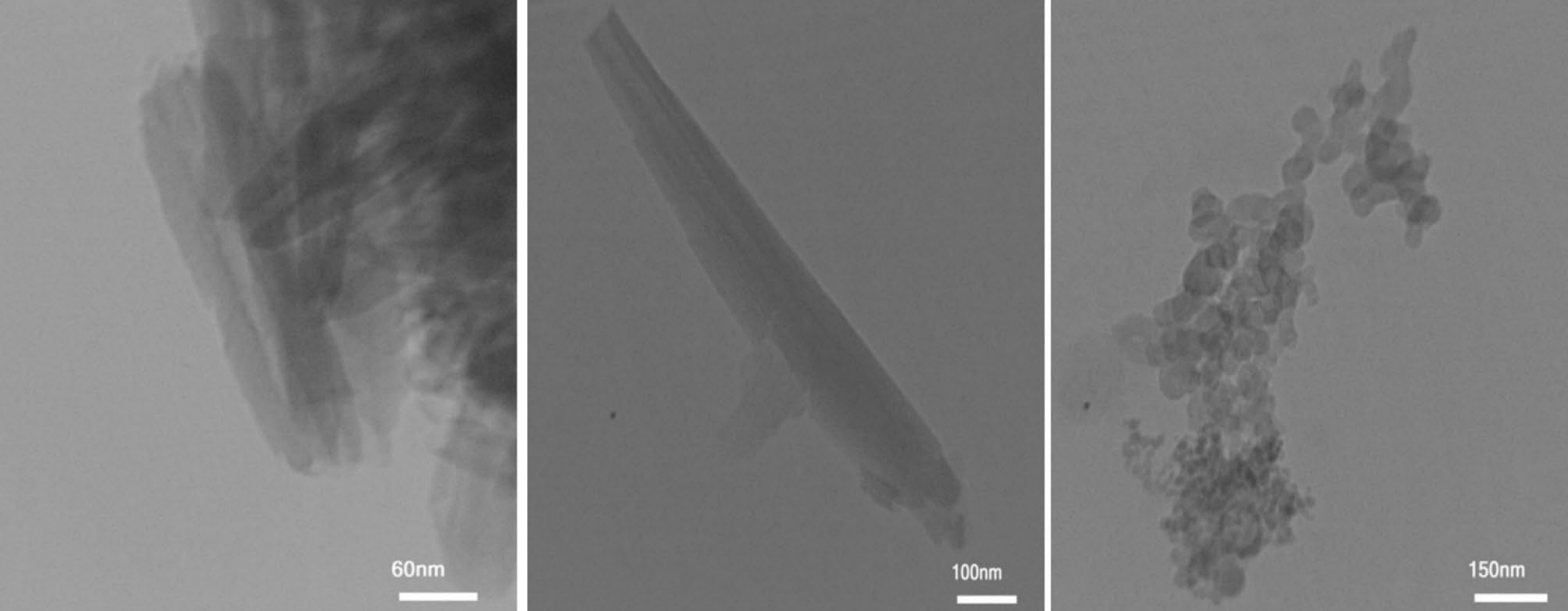
TEM images of Pd@Hal-DAB-PC.

The surface structure and morphology of the Pd@Hal-DAB-PC nanocatalyst were further studied using TEM. The TEM images of the Pd@Hal-DAB-PC displayed the tubular morphology of Hal. Besides, Pd NPs can be seen on the surface of the support, confirming immobilization of Pd NPs (Fig. 4).

**Figure 5:**
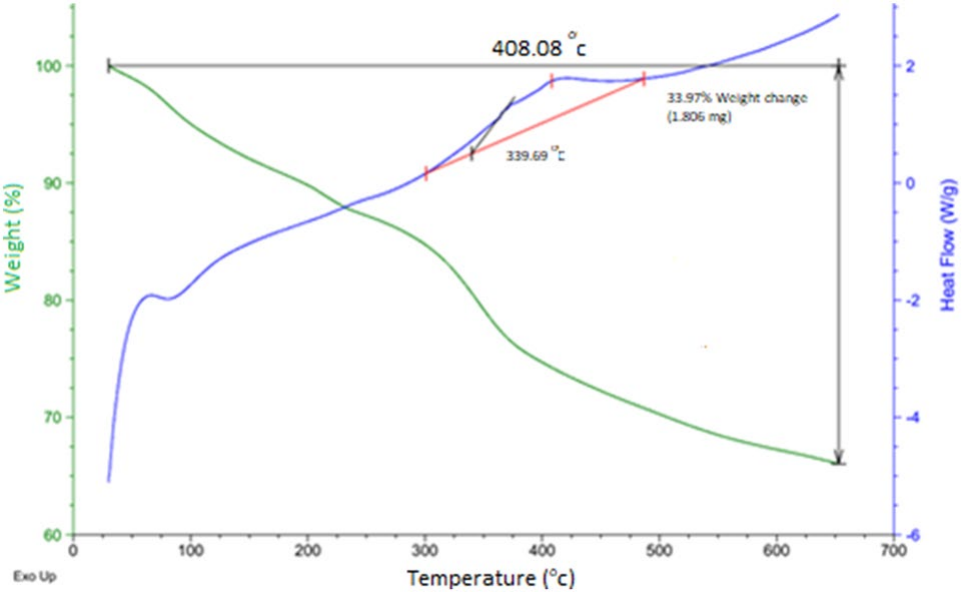
The TGA curve of Pd@Hal-DAB-PC.

In the following, the thermostability of the catalyst and the percentage of the organic groups linked to the surface of the Hal were examined using TGA. The thermograph of the Pd@Hal-DAB-PC depicts three decomposition steps (Fig. 5). The initial weight reduction (about 10–12%) at low temperatures (70–120 °C) can be related to the removal of the adsorbed water and surface hydroxyl groups. A higher weight loss (10–20%) in the region 330–410 °C corresponds to the decomposition of the Schiff base segment and the chloropropyl groups grafted to the Hal. The next minimal weight loss is due to the elimination of chemisorbed water. The observed total weight reduction is 33.97 until 700 °C. These results show good thermal stability of Pd@Hal-DAB-PC.

**Figure 6:**
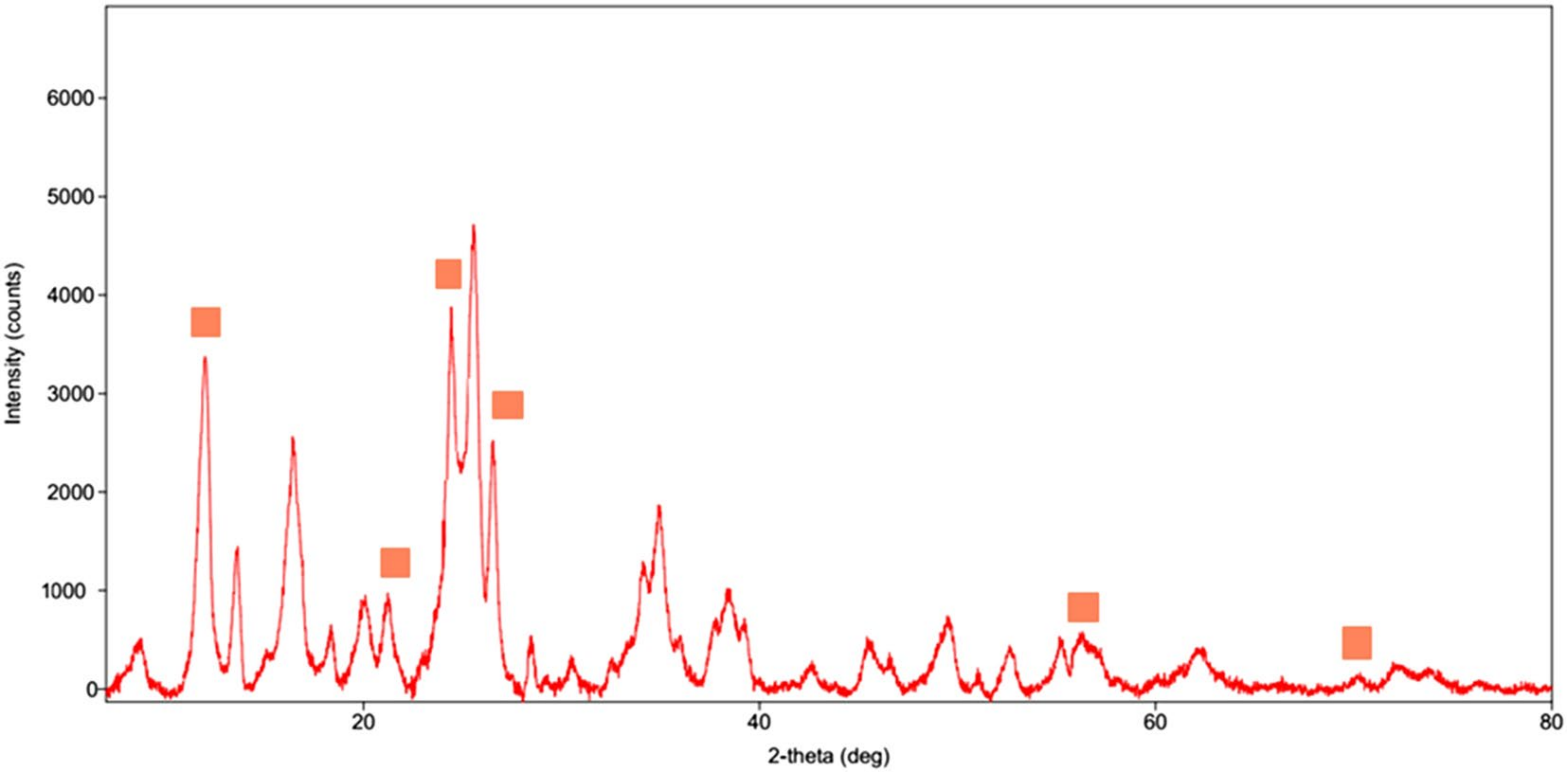
The XRD curve of Pd@Hal-DAB-PC.

To obtain information about the crystalline nature and phase composition of the Pd@Hal-DAB-PC, X-ray powder diffraction (XRD) analysis was accomplished. The XRD curve of the Pd@Hal-DAB-PC (Fig. 6) reveals the distinctive peaks at 2θ = 8°, 12°, 22.6°, 28.2°, 31.5°, 57°, and 68° which are in good record with the XRD pattern of Hal (JCPDS card no. 29-1487)^33,34^. This result confirms that the tubular structure of Hal does not destroy during the functionalization and stabilization of Pd. Also, the diffraction peaks of Pd NPs were not observed in the sample, affirming the high dispersion of Pd NPs as well as their low loading on the support^35^. This result was confirmed by ICP analysis and the loading of Pd was found to be 0.85 wt%.

### Catalytic activity

After the successful synthesis and identification of Pd@Hal-DAB-PC, its catalytic behavior was evaluated in the Sonogashira coupling reaction. For this purpose, the coupling of iodobenzene with phenylacetylene was chosen as a probe reaction and the efficient parameters on this reaction like catalyst amount, temperature, and type of solvent and base were optimized to achieve the highest product yield. The results were summarized in Table 1. K_2_CO_3_ as a base in the presence of H_2_O as a green solvent and 10 mol% of Pd@Hal-DAB-PC at 80 °C was found to be the best reaction conditions.

**Table 1:** Optimization for the coupling of aryl iodide and phenyl acetylene^a^.

Entry	Reaction condition	Catalyst (mol%)	Base	Time (h)	Yield (%)
**1**	H_2_O/r.t	10	K_2_CO_3_	3	90
**2**	H_2_O/50 °C	10	K_2_CO_3_	2.5	92
**3**	H_2_O/80 °C	10	K_2_CO_3_	2	97
**4**	H_2_O/reflux	10	K_2_CO_3_	2	95
**5**	EtOH, r.t	10	K_2_CO_3_	3	82
**6**	EtOH, reflux	10	K_2_CO_3_	2.5	88
**7**	CH_3_CN, reflux	10	K_2_CO_3_	3	85
**8**	CHCl_3_, reflux	10	K_2_CO_3_	3.2	80
**9**	Toluene, 80 °C	10	K_2_CO_3_	2.8	79
**10**	H_2_O/80 °C	15	K_2_CO_3_	2	96
**11**	H_2_O/80 °C	5	K_2_CO_3_	2.5	82
**12**	H_2_O/80 °C	10	NaOH	2.5	84

^a^Reaction conditions: iodobenzene (1 mmol), phenylacetylene (1.2 mmol), base (3 mmol), and solvent (5 ml).

In the next step, the wide utilization of Pd@Hal-DAB-PC was further examined (Table 2). A broad set of aryl iodides involving electron-withdrawing, electron-donating, and sterically hindered groups effectively reacted with terminal alkynes to furnish the respective products in high yields. All compounds are known and their physical data were compared and validated with those of authentic samples. Some selected spectral data are presented in supporting information (Figures S1–S10).

**Table 2:**
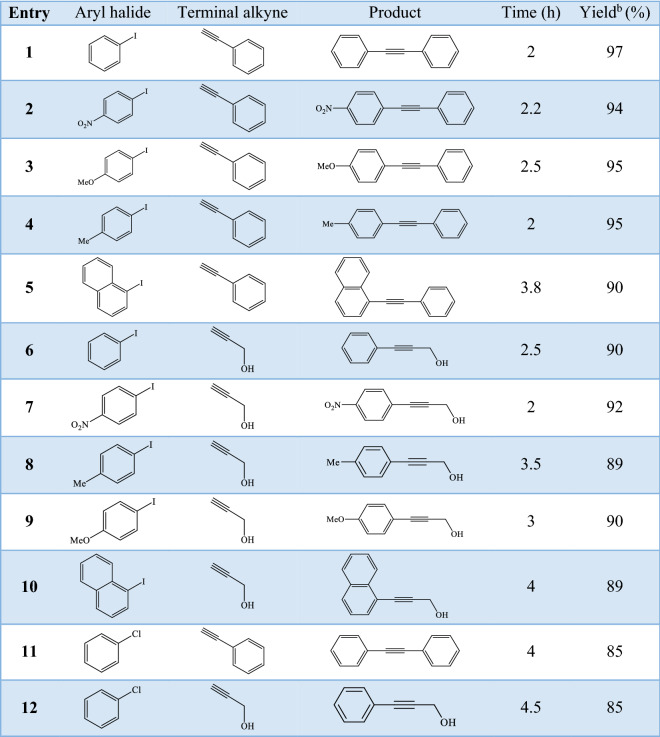
Pd@Hal-DAB-PC catalyzed Sonogashira reaction of various halides with terminal alkynes^a^.

^a^Reaction condition: aryl halide (1.0 mmol), terminal alkyne (1.2 mmol), Pd@Hal-DAB-PC (10 mol%) and K_2_CO_3_ (3.0 mmol) in H_2_O at 80 °C.

Interestingly, good yields of products are also provided in the coupling of less-reactive (more challenging) aryl chloride with various terminal alkynes.

### Mechanism

**Scheme 3:**
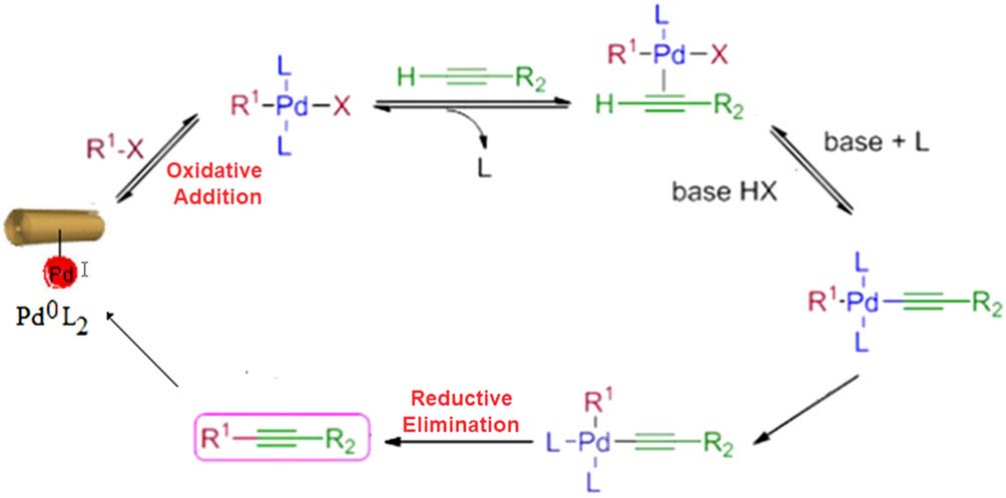
Plausible reaction mechanism.

A reasonable mechanistic pathway was suggested as depicted in Scheme 3. Accordingly, initial oxidative addition of [Pd(0)L_2_] to the aryl or vinyl halide takes place which is followed by reversible coordination of the alkyne, creating an alkyne-Pd(II) complex. Then, in the presence of a base, it was deprotonated with concurrent coordination of the acetylene ligand to the metal. Next, upon reductive elimination, the [Pd(II)R^1^(C[tbond]CR^2^)L_2_] complex, releases the cross-coupled product along with regeneration of the catalyst species, [Pd(0)L_2_].

### Reusability of Pd@Hal-DAB-PC

**Figure 7:**
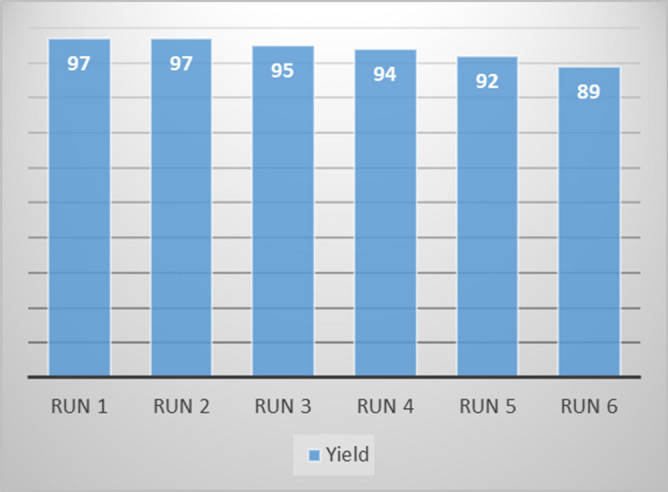
Reusability of Pd@Hal-DAB-PC.

The stability/reusability of a catalyst is a critical factor from the economic and industrial points of view. In this context, the stability of Pd@Hal-DAB-PC was inspected in the model reaction under the optimal conditions (Fig. 7). After completion of the reaction in each run, the catalyst was collected via centrifugation, washed with ethanol, and then reused in the next cycle. The recycling experiments show that Pd@Hal-DAB-PC is highly resistant with a slight decrease in its catalytic efficiency during six runs.

**Figure 8:**
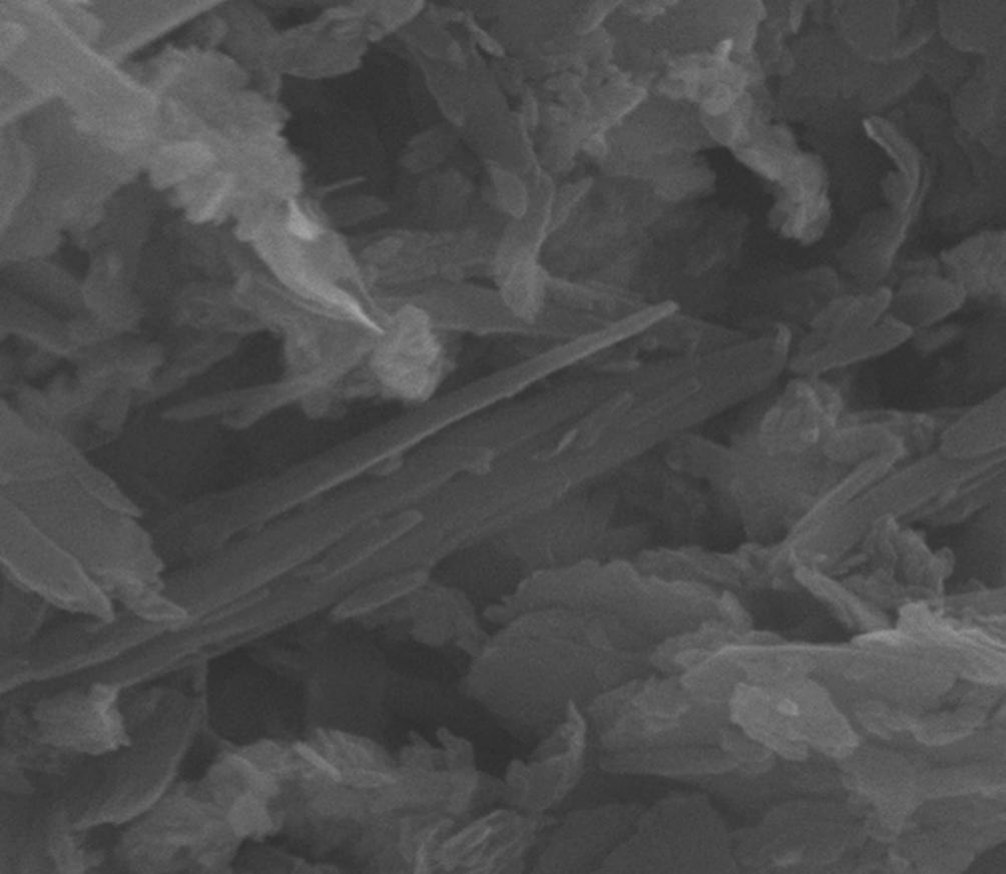
SEM image of the recycled catalyst after six reaction runs.

To examine any effect on the catalyst morphology during the recyclization process, after six reaction runs, the recycled catalyst was submitted to the SEM analysis, (Fig. 8). As illustrated in Fig. 8, the SEM image of the recycled catalyst was compared with the freshly used catalyst and was found to be similar.

## Conclusions

In summary, we reported the fabrication of Pd@Hal-DAB-PC as a new hybrid catalyst by functionalizing Cl-Hal with Schiff base and the next stabilization of Pd NPs. The obtained catalyst was capable to successfully catalyze the Sonogashira cross-coupling reactions in aqueous media under ligand- and Cu-free reaction conditions. Furthermore, Pd@Hal-DAB-PC was stable and could be reused for six runs while its efficiency was largely maintained.

## Supplementary Information

Supplementary Information

## Data Availability

The raw/processed data that supports the findings of this study are available from the corresponding author upon reasonable request.
