# Supplementary material for: Pd NPs supported on halloysite functionalized with Schiff base as an efficient catalyst for Sonogashira reaction
Source: Sci Rep. 2021 Mar 18;11:6223. doi: 10.1038/s41598-021-85821-2 (PMC7973577; doi:10.1038/s41598-021-85821-2)
Supplement: Supplementary file 1 — Supplementary Information [file 41598_2021_85821_MOESM1_ESM.pdf]

Supporting information

Pd NPs supported on halloysite functionalized with Schiff base as an efficient catalyst for Sonogashira reaction

Mansoureh Daraie[1*], *Majid M. Heravi*[1*], *Yalda Rangraz*[1], *Zahra Besharati*[1]

[1]*Department of Chemistry, School of Science, Alzahra University, Vanak, Tehran, Iran.*

[*]Corresponding author: Majid M. Heravi (m.heravi@alzahra.ac.ir ; mmh1331@yahoo.com)

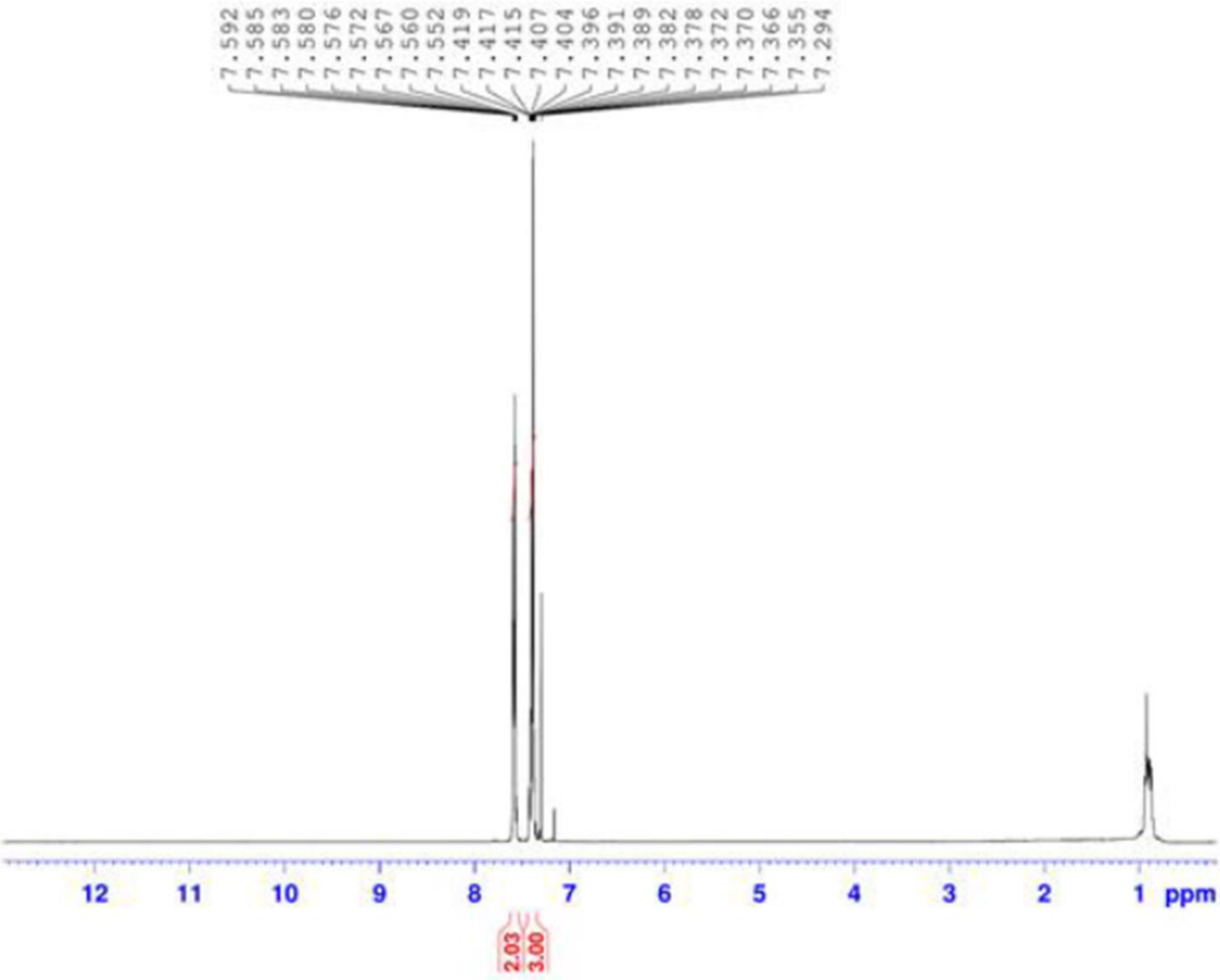

Figure S1. ^{1}H NMR of 1,2-diphenylethyne

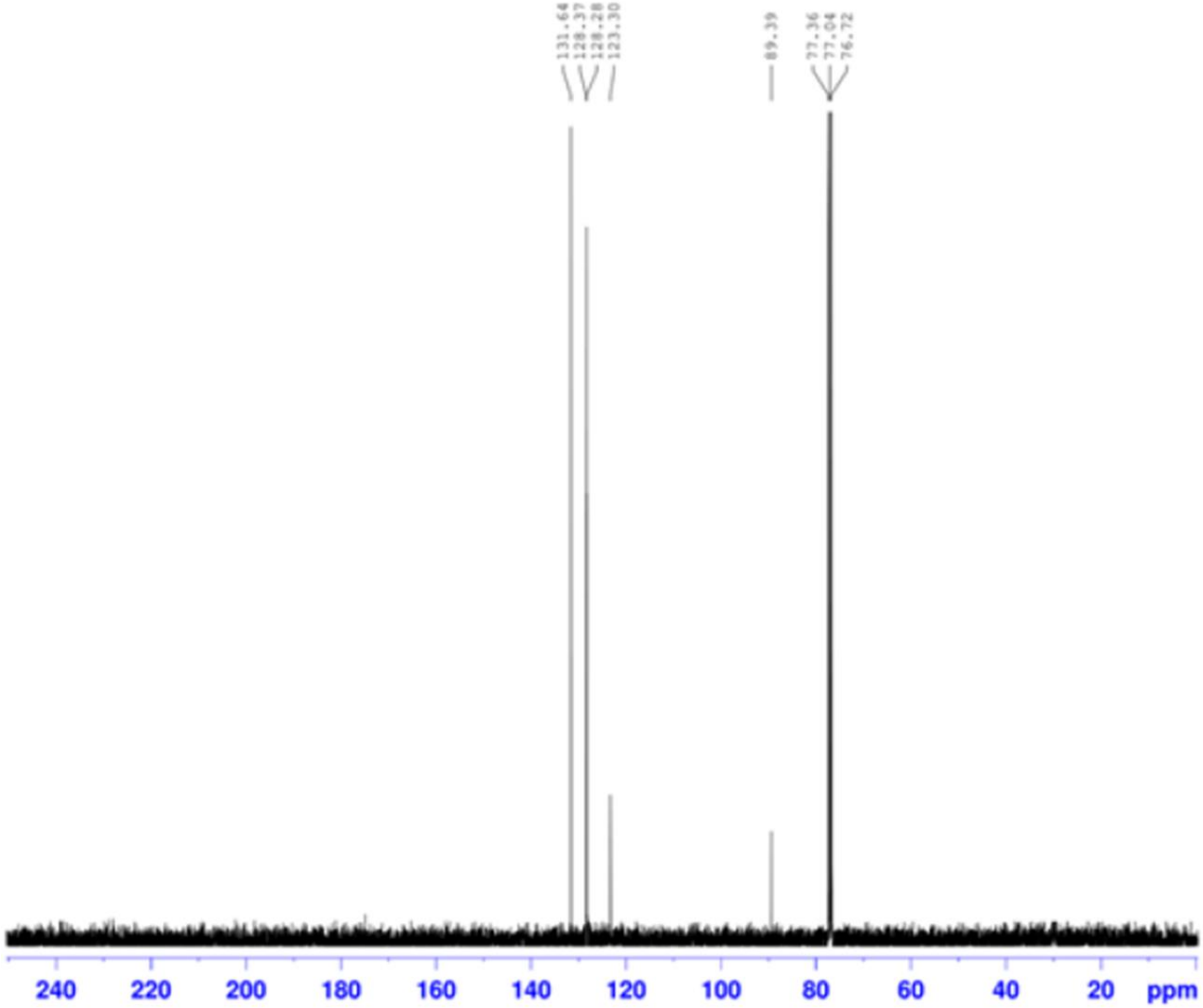

Figure S2. ^{13}**C NMR of 1,2-diphenylethyne**

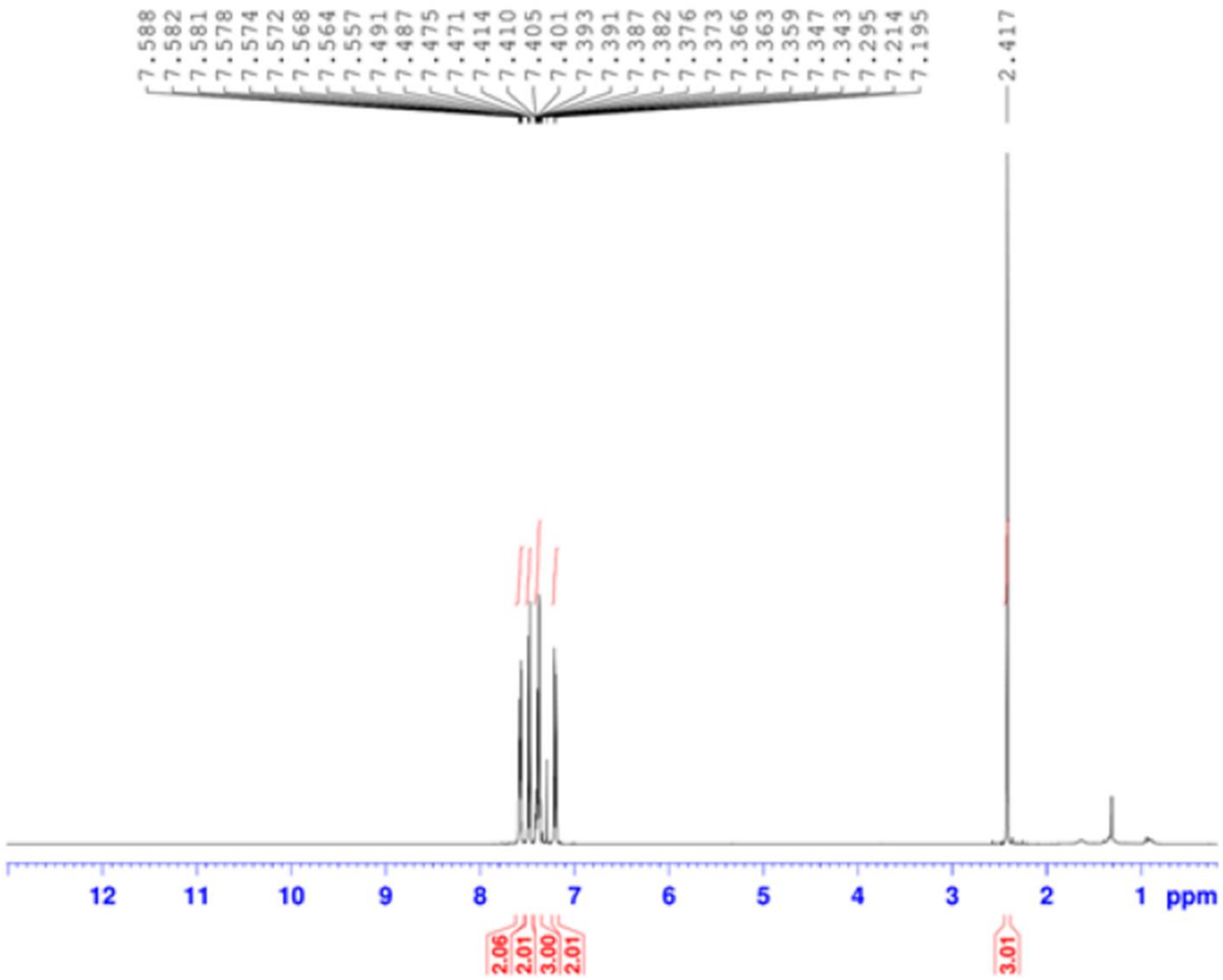

Figure S3. ^{1}H NMR of 1-methyl-4-(phenylethynyl)benzene

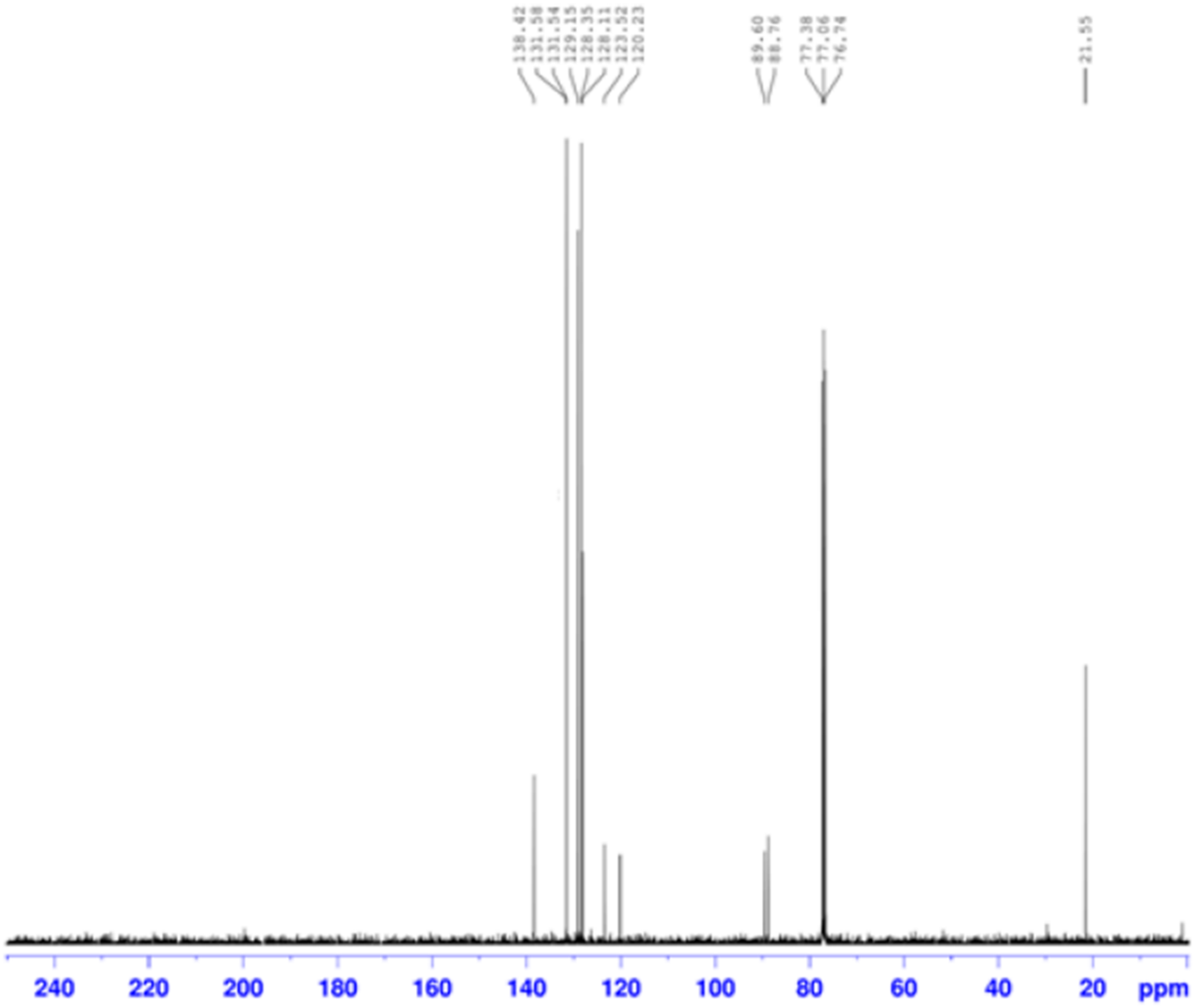

Figure S4. ^{13}**C NMR of 1-methyl-4-(phenylethynyl)benzene**

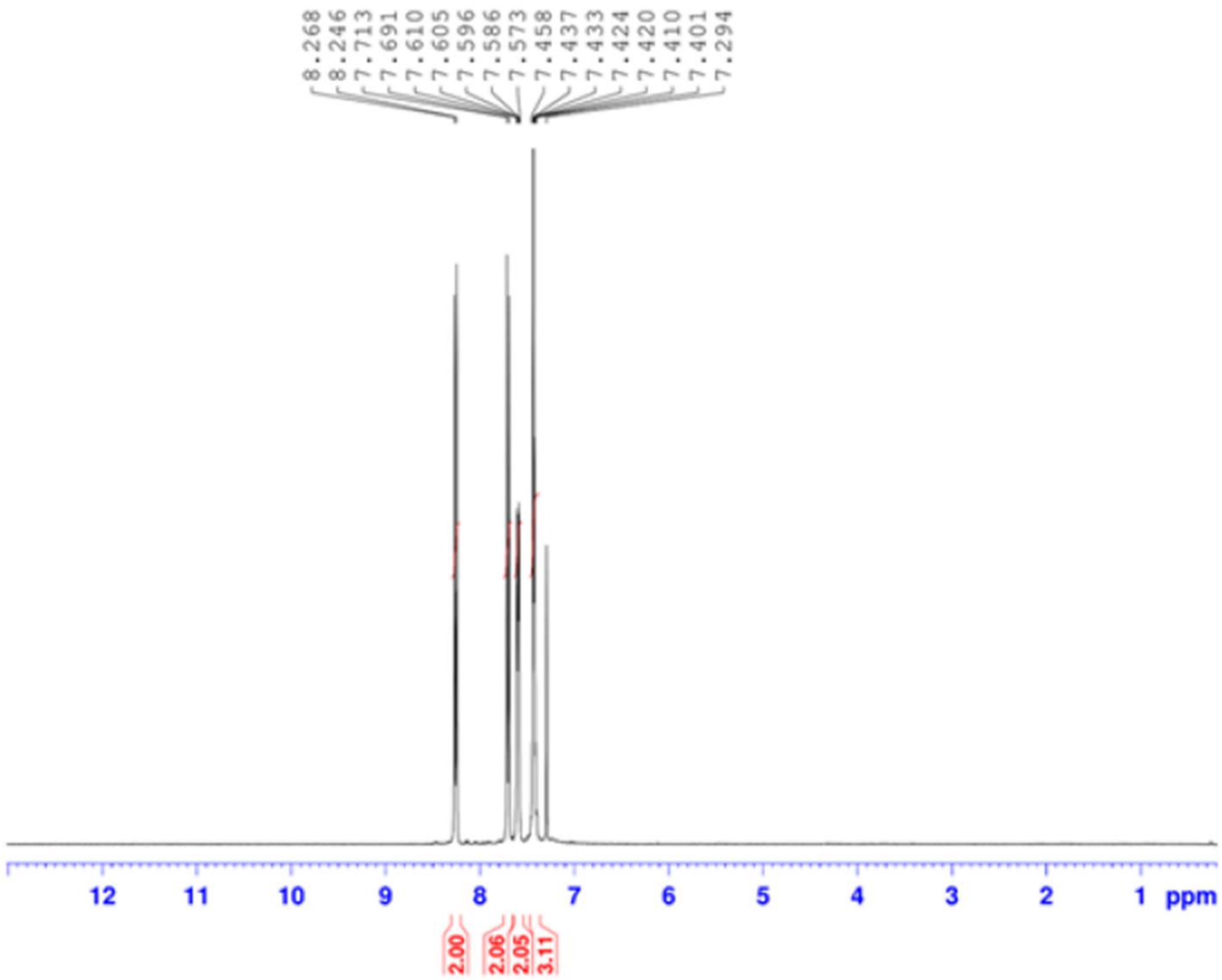

Figure S5. ^{1}H NMR of 1-nitro-4-(phenylethynyl) benzene

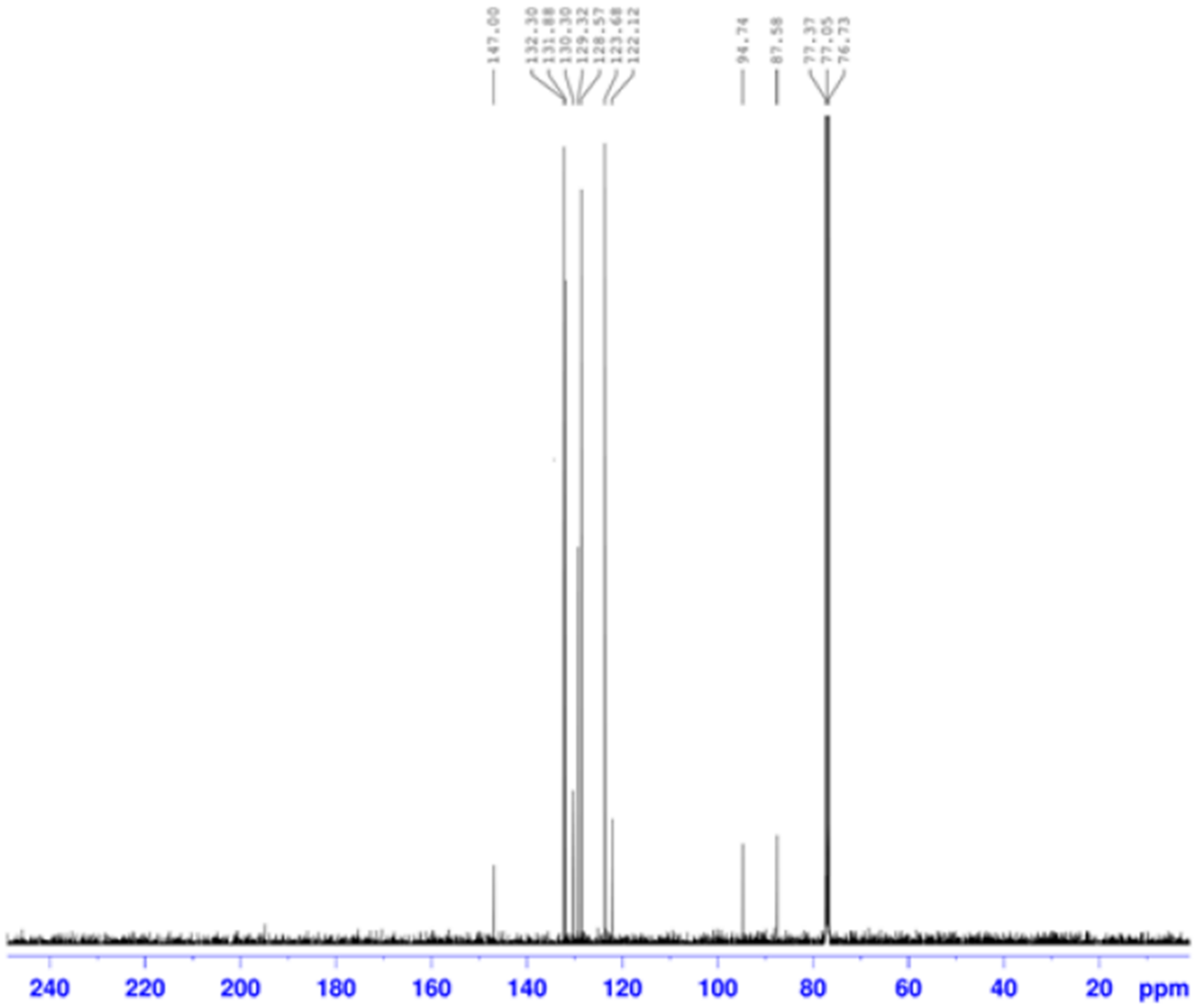

Figure S6. ^{13}C NMR of 1-nitro-4-(phenylethynyl) benzene

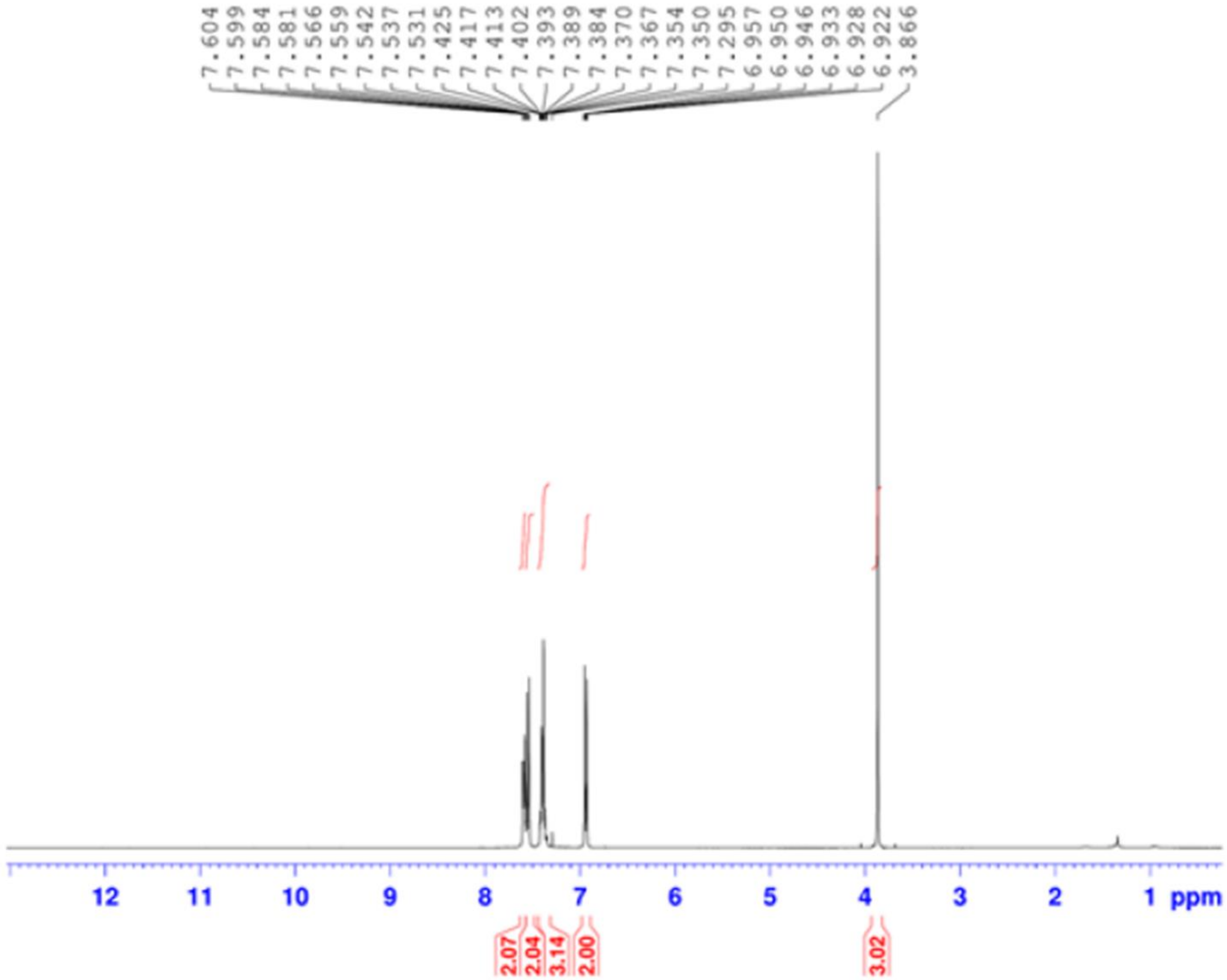

Figure S7. ^{1}H NMR of 1-methoxy-4-(phenylethynyl)benzene

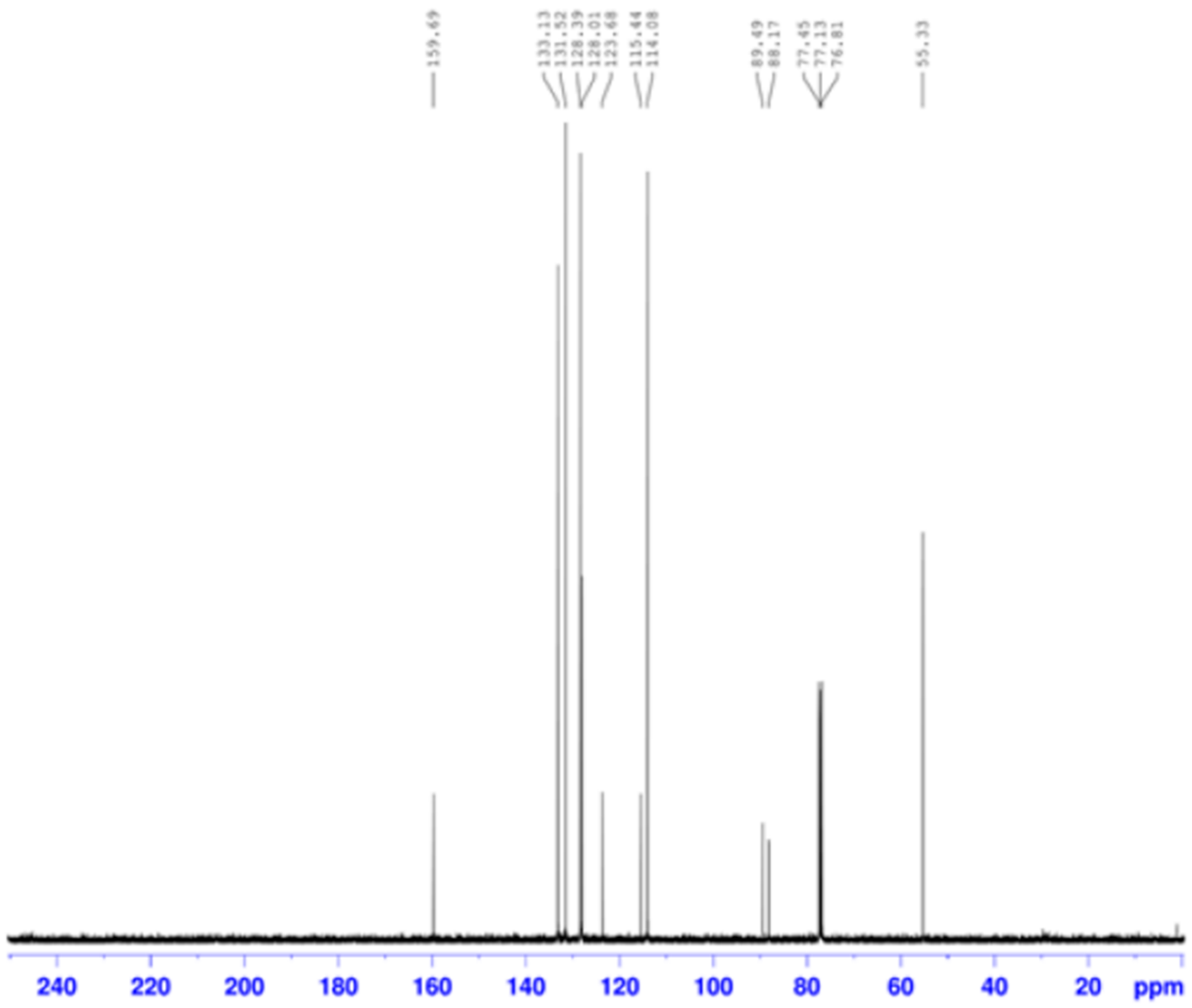

Figure S8. ^{13}C **NMR of 1-methoxy-4-(phenylethynyl)benzene**

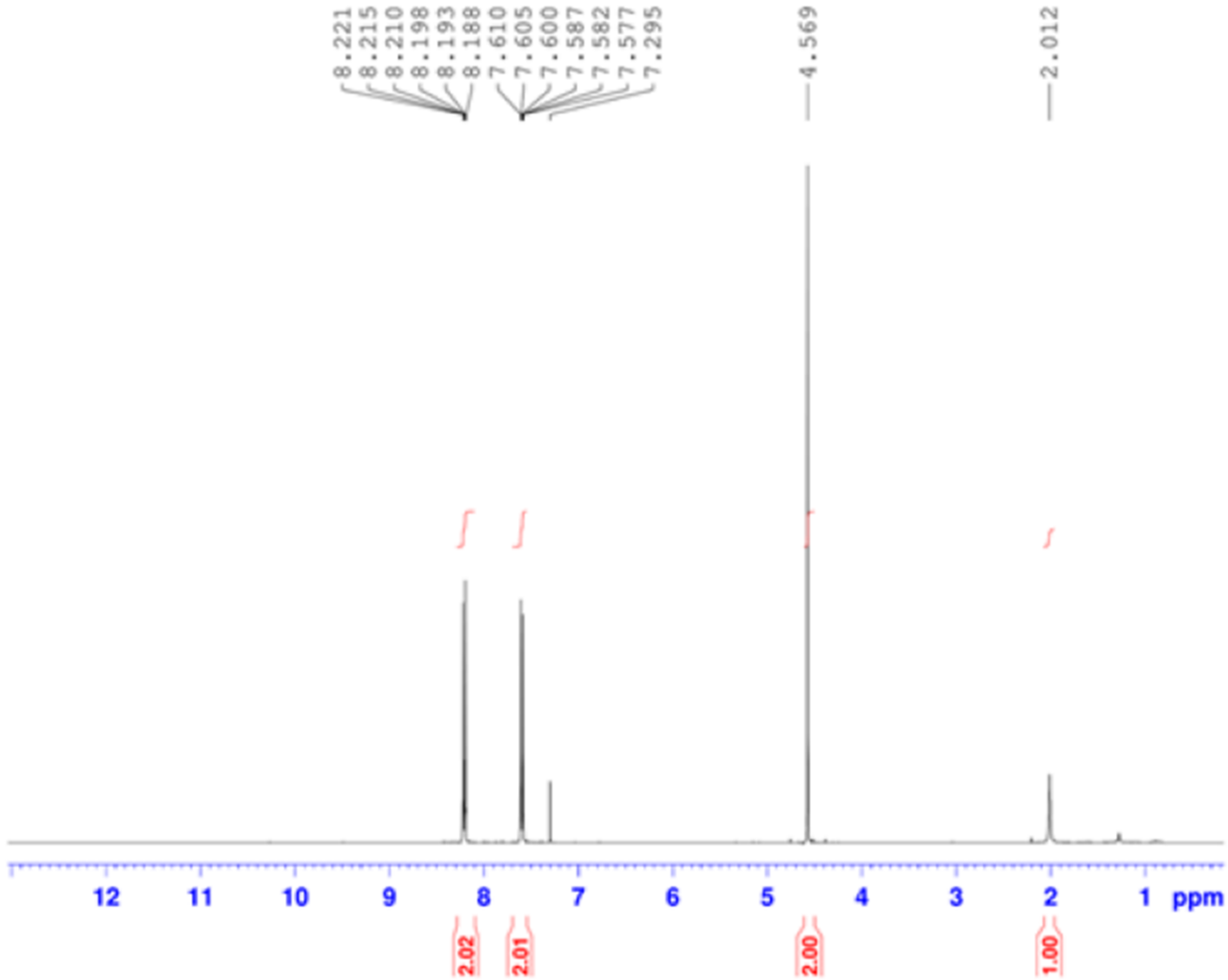

Figure S9. [1]**H NMR of 3-(4-nitrophenyl)prop-2-yn-1-ol**

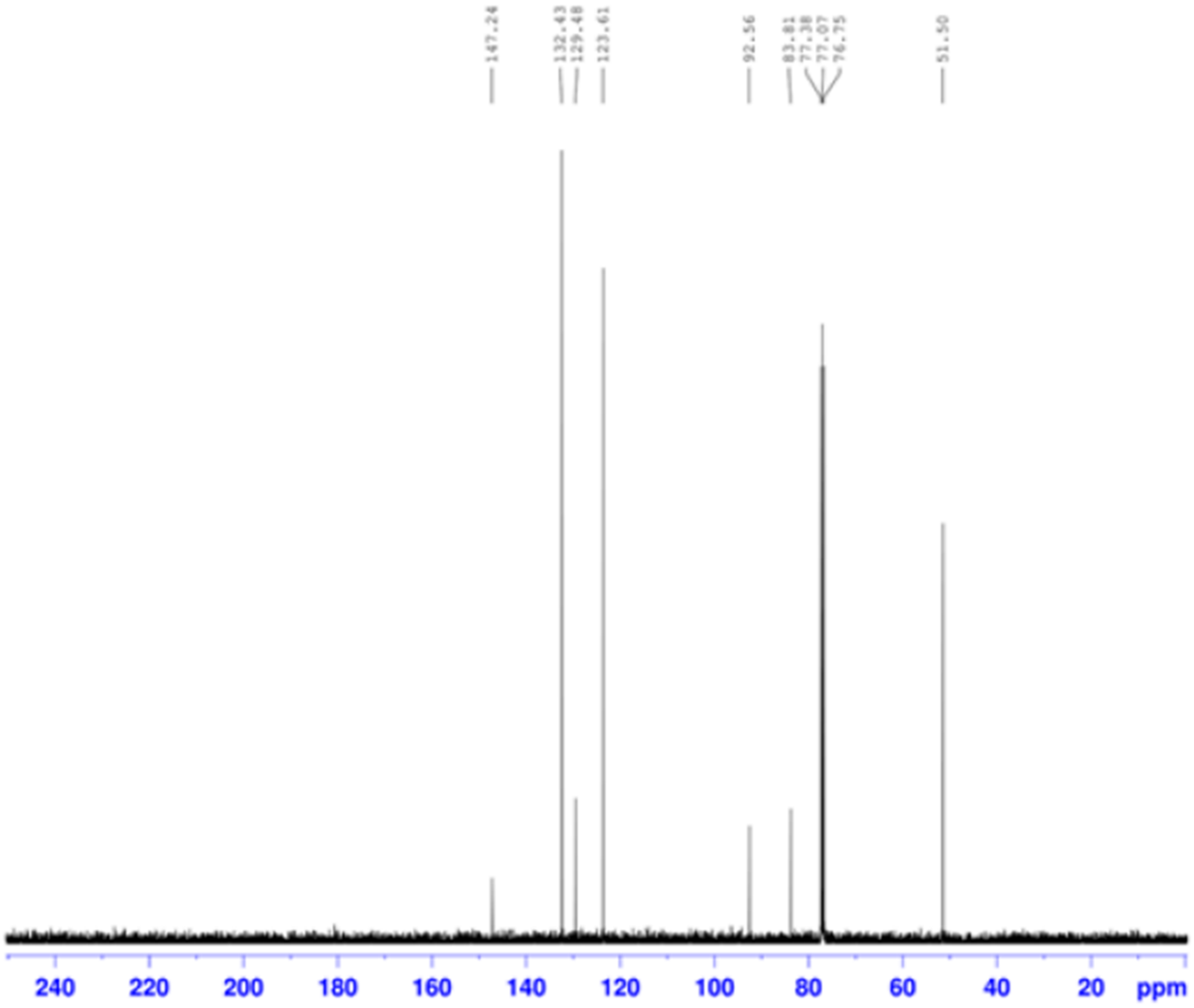

Figure S10. ^{13}C **NMR of 3-(4-nitrophenyl)prop-2-yn-1-ol**
